# Prospero and Pax2 combinatorially control neural cell fate decisions by modulating Ras- and Notch-dependent signaling

**DOI:** 10.1186/1749-8104-6-20

**Published:** 2011-05-03

**Authors:** Mark Charlton-Perkins, S Leigh Whitaker, Yueyang Fei, Baotong Xie, David Li-Kroeger, Brian Gebelein, Tiffany Cook

**Affiliations:** 1 Department of Pediatric Ophthalmology, Cincinnati Children's Hospital Medical Center, 3333 Burnet Ave, Cincinnati, OH 45229, USA; 2 Division of Developmental Biology, Cincinnati Children's Hospital Medical Center, 3333 Burnet Ave, Cincinnati, OH 45229, USA; 3 Molecular and Developmental Biology Graduate Program, University of Cincinnati, Cincinnati Children's Hospital Medical Center, 3333 Burnet Ave, Cincinnati, OH 45229, USA

## Abstract

**Background:**

The concept of an equivalence group, a cluster of cells with equal potential to adopt the same specific fate, has served as a useful paradigm to understand neural cell type specification. In the *Drosophila *eye, a set of five cells, called the 'R7 equivalence group', generates a single photoreceptor neuron and four lens-secreting epithelial cells. This choice between neuronal versus non-neuronal cell fates rests on differential requirements for, and cross-talk between, Notch/Delta- and Ras/mitogen-activated protein kinase (MAPK)-dependent signaling pathways. However, many questions remain unanswered related to how downstream events of these two signaling pathways mediate distinct cell fate decisions.

**Results:**

Here, we demonstrate that two direct downstream targets of Ras and Notch signaling, the transcription factors Prospero and dPax2, are essential regulators of neuronal versus non-neuronal cell fate decisions in the R7 equivalence group. Prospero controls high activated MAPK levels required for neuronal fate, whereas dPax2 represses Delta expression to prevent neuronal fate. Importantly, activity from both factors is required for proper cell fate decisions to occur.

**Conclusions:**

These data demonstrate that Ras and Notch signaling are integrated during cell fate decisions within the R7 equivalence group through the combinatorial and opposing activities of Pros and dPax2. Our study provides one of the first examples of how the differential expression and synergistic roles of two independent transcription factors determine cell fate within an equivalence group. Since the integration of Ras and Notch signaling is associated with many developmental and cancer models, these findings should provide new insights into how cell specificity is achieved by ubiquitously used signaling pathways in diverse biological contexts.

## Background

A remarkably small number of signaling cascades are used across phyla to control cell specification. Thus, specificity must arise from combinatorial and cross-regulatory interactions among these pathways. Significant progress has been made in identifying the common immediate effectors for many of these conserved pathways, including those used during Ras- and Notch-dependent signaling. However, how these common pathways drive distinct cell fate choices in particular organs remains unclear.

Many of the common components within the Ras/mitogen-activated protein kinase (MAPK) and Notch/Delta signaling pathways have been identified and studied extensively in the developing *Drosophila *compound eye [1-4]. This is in large part because each of the 20 cells present in the approximately 750 repeating adult eye units (ommatidia) in this organ is stereotypically and sequentially recruited by the reiterative use of these two pathways. First, the R8 photoreceptor neuron is selected through Notch-dependent lateral inhibition, followed by stepwise recruitment of the R2/R5, R3/R4, R1/R6, and R7 photoreceptors (PRs), four cone cells (CCs), two primary pigment cells (PPCs), and approximately nine shared highly pigmented interommatidial cells by use of both Ras and Notch signaling [5-9]. How Ras and Notch are differentially interpreted in these different cell types, however, is not understood.

Specification of the R7 photoreceptor provides a particularly useful model for exploring Ras and Notch signal integration in the fly eye. The R7 and CCs are the last cells to be recruited to ommatidial clusters before pupation, and classic studies have established that prior to their specification, these five cells comprise an 'R7 equivalence group' [10,11]. All cells within the R7 equivalence group express Notch, the epidermal growth factor (EGF) receptor (EGFR), and Sevenless, a tyrosine kinase receptor that, like EGFR, signals via the Ras/MAPK pathway. The ligands Delta and EGF are made available from the previously specified PRs R1 and R6, but only one cell can directly contact Boss, the Sev ligand, present on the surface of the R8 PR [12-15]. This cell, receiving higher Ras/MAPK levels, is driven towards an R7 fate, while the remaining cells that do not receive Sev signaling become epithelial CCs [2,16,17]. These studies have led to the prevailing model that Ras/MAPK signaling is necessary and sufficient for neuronal versus non-neuronal cell fate decisions in this group of cells. However, more recent work in the fly eye has shown that Ras/MAPK levels also indirectly control Delta expression [18,19], a neurogenic factor in much of the developing nervous system [20], and mutants affecting Ras or Notch signaling cause only partial R7 versus CC fate switches [2,10,12,17,19,21-24]. Thus, it is important to clarify the role of these signaling pathways in R7 versus CC fate choices, as it serves as a useful model for addressing how different levels of signaling mediate cell-specific fate decisions during development.

Two specific downstream targets of Ras and Notch within the R7 equivalence group are the transcription factors Prospero (Pros) and dPax2 (also known as *sparkling *(*spa*) or *shaven *(*Sv*)) [25]. Pros is expressed in all five cells and is later up-regulated in the presumptive R7 by Sevenless [26,27]. dPax2, on the other hand, is restricted to the four CC precursor cells, and is later expanded into the PPCs, the next cells to form in the eye [28]. Enhancers partially reflecting these Pros and dPax2 expression patterns have been identified. Pros expression is directly up-regulated by the EGF/Ras/MAPK-activated ETS transcription factor PntP2, and is probably indirectly regulated by Notch signaling [26,27,29]. dPax2, instead, is controlled by the combined direct inputs of PntP2 and the Notch/Su(H) activation complex [30,31]. While these studies provide evidence that Pros and dPax2 are direct transcriptional targets of Ras and Notch signaling, the functional relevance of this regulation remains an unanswered question.

Here, we demonstrate that Pros and dPax2 are expressed at different levels in distinct subtypes of CC precursors, indicating that not all CCs are 'equivalent'. Further, we show that individual mutations in Pros or Pax2 cause minor changes in CC numbers, while their simultaneous removal prevents all CC recruitment, revealing that these factors combinatorially control CC fate determination. Mechanistically, we demonstrate that Pros maintains high-activated MAPK (pERK) levels in the R7 equivalence group, thus promoting neuronal PR fate within this cell population. Conversely, dPax2 transcriptionally suppresses Delta expression in CC precursors, serving as an anti-neuronal factor. Importantly, the integration of Pros and dPax2 functions via Ras and Notch signaling feedback is necessary to fully control neuronal versus non-neuronal cell fate decisions in the fly eye. Since Pros and dPax2 orthologs are co-expressed in many developmental systems requiring Ras/MAPK and Delta/Notch inputs, it is likely that these processes are evolutionarily conserved.

## Results

### Pros and dPax2 genetically interact to recruit CCs and control lens formation

Pros is known to regulate late aspects of R7 terminal differentiation [26,32,33], while dPax2 has been reported to be important for CC shape and organization and PPC recruitment during pupal development [28,34]. However, the early expression of these factors in response to signals required for R7 versus CC specification suggests that they also may participate in cell fate decisions within the R7 equivalence group.

**Figure 1:**
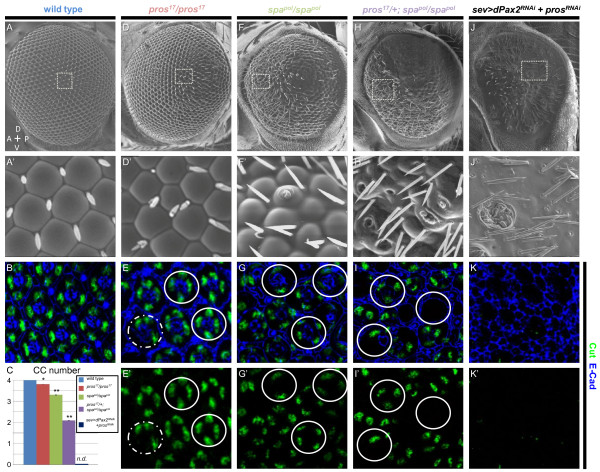
**Pros and dPax2 together control cone cell development and lens formation**. **(A,B,D-K) **Scanning electron micrographs (SEMs) of adult eyes (A,D,F,H,J) or immunostaining of 45% pupal retinas for the CC-specific marker Cut (green) and the membrane marker E-cadherin (blue) (B,E,G,I,K) were analyzed from the following genotypes: *yw^67^*; Sp/CyO; TM2/TM6B (A,B), *ey^flp3.5^*; Sp/CyO; FRT82*ubi*-GFPnls, RpS3/FRT82B-*pros*^17 ^(D,E), *yw^67^*; Sp/CyO; TM2/TM6B; *spa^pol^*/*spa^pol^*(F,G), *yw^67^*; Sp/CyO; FRT82B-*pros*^17^/TM6B; *spa^pol^*/*spa^pol^*(H,I), and UAS-*d*Pax2^RNAi^; sev-GAL4/CyO; UAS-Pros ^RNAi^/TM6B (J,K). A slightly more severe phenotype from that shown in (J,K) is apparent in *pros*^17^/*spa^pol^*double mutants, but these eyes collapsed during SEM and were particularly difficult to dissect. Panels (A',D',F',H',J') represent the boxed areas in the corresponding larger SEM. Circles represent CC clusters within individual ommatidia, and the dotted circle in (E) represents a rare yet significant loss of one CC observed in *pros *mutants. Control eyes have regular lens facets by SEM (A) and four CCs per ommatida by Cut and E-cadherin double-staining (B). **(C) **Quantification of CC numbers shows a strong genetic interaction and dual requirement for Pros and dPax2 in CC specification. *P < 0.05, **P < 0.001, n.d. = none detected. Error bars represent standard deviation. (D,E) *pros^17^*Minute LOF eyes (see Materials and methods) show mild roughening by SEM (D), and a rare yet significant loss of one CC (E, dotted circle). (F,G) *spa^pol^*eyes show some roughening (F), consistent with a regular loss in PPCs [28] and at least one CC (G,G', circles). Removing one copy of *pros *from *spa^pol^*mutants causes further perturbation of lens morphogenesis, with holes in the center of the lenses frequently observed (H), and CC number is reduced to approximately two per ommatidia (I). *sev>*dPax2^RNAi ^+ ProsR^RNAi ^lack lens facets by SEM (J) and Cut-positive cells are not detected (K).

CCs, together with PPCs, secrete highly regular corneal lenses, a feature easily observable by scanning electron microscopy (SEM) of adult flies (Figure 1A) [5]. Therefore, to rapidly assess potential roles for Pros and/or Pax2 in CC recruitment, we examined corneal lens formation in different mutant combinations. Removing Pros function from the majority of the eye using Minute clones of the *pros^17^*null allele (see Materials and methods; Figure 1D) or by eye-specific RNA interference (RNAi)-mediated knockdown (data not shown) leads to a relatively minor phenotype, with lenses having only slightly irregular shapes and sizes (Figure 1D) compared with wild-type eyes (Figure 1A). Similar results are obtained by reducing dPax2 function using an eye-specific *dPax2 *mutant, *spa^pol^*(Figure 1F,F'), or dPax2 RNAi knockdown (data not shown), although lenses in both *dPax2 *genetic backgrounds become progressively less defined posteriorly. In marked contrast, *dPax2 *mutants lacking one copy of *pros *show a much more severe lens phenotype than either individual mutant, with many ommatidia having reduced, defective, or missing lenses (Figure 1H); and full removal of both Pros and dPax2 leads to total loss of lens structures (Figure 1J). These data indicate that alone, Pros and dPax2 have somewhat subtle effects on lens formation, but together, genetically interact to drive all of lens formation.

To verify whether the external adult phenotypes observed by SEM associate with changes in CC recruitment, we analyzed the expression of a CC-specific marker, Cut, in pupal retinas from various *pros*/*dPax2 *mutant backgrounds. Similar to wild-type retinas (Figure 1B), a normal complement of four CCs is present in the center of large *pros *mitotic clones (Figure 1E, solid circles), although occasional ommatidia with only three CCs are observed (Figure 1E, dotted circle) (see Table 1 for quantification). This phenotype is consistent with the subtle changes in lens morphology observed in *pros *mutants (Figure 1D). We note, though, that at the edges of clones and in highly mosaic *pros *tissue, ommatidia with irregular numbers of CCs frequently develop (Additional file 1A,B), suggesting that Pros may contribute to non-cell autonomous events during CC recruitment (see Discussion). In *dPax2 *mutants, we detect Cut expression in all CCs in the pupal retina, similar to previous reports [28]. In contrast to previous studies, however, we find a consistent and significant loss of at least one CC per ommatidia in these mutants (Figure 1C,G). In agreement with the SEM results (Figure 1H,J), removing a single copy of *pros *from *dPax2 *loss-of-function eyes reduces CC number to two CCs per ommatidia (Figure 1C,I), and in *pros*/*dPax2 *double loss-of-function mutants, no CCs are detected (Figure 1C,K). Combined, these experiments demonstrate that Pros and dPax2 individually affect the specification of a limited number of CCs, but that they synergistically recruit the full complement of CCs, thus controlling lens morphogenesis.

**Table 1:** Summary of photoreceptor and cone cell quantifications from Pros and dPax2 genetic manipulations

Genotype	Mean number	n	Standard deviation	Significance
CC quantification				
*pros^17^/pros^17^*	3.83	129	0.04	**
*spa^pol^/spa^pol^*	3.14	126	3.31	**
*pros^17 ^/+;spa^pol ^/spa^pol^*	2.10	109	0.01	**
*sev>dPax2^RNAi ^+ pros^RNAi^*	ND	-	-	-
sev>dPax2	4.15	129	0.05	*
sev>Pros	4.54	114	0.15	**
sev>Pros + dPax2	5.08	121	0.09	**
*sev*>Pros; *spa^pol^/spa^pol^*	0.00	143	0.00	-
*sev*>dPax2 + *pros^RNAi^*	4.65	103	0.06	**
				
PR quantification				
*pros^17^/pros^17^*	7.93	129	0.03	NS
*spa^pol^/spa^pol^*	7.95	126	0.00	NS
sev>dPax2	7.81	129	0.03	**
sev>Pros	8.15	114	0.06	**
*sev*-Ras^v12^	8.65	202	0.07	**
sev>Pros + dPax2	8.02	121	0.01	NS
*sev*>Pros; *spa^pol^/spa^pol^*	11.00	143	0.03	**
*sev*>dPax2 + *pros^RNAi^*	7.22	103	0.05	**

Statistical differences from wild-type numbers were analyzed by one-way ANOVA. *P < 0.05, **P < 0.001; NS, not significant; ND, not determined.

### Prospero and dPax2 define distinct cone cell populations

**Figure 2:**
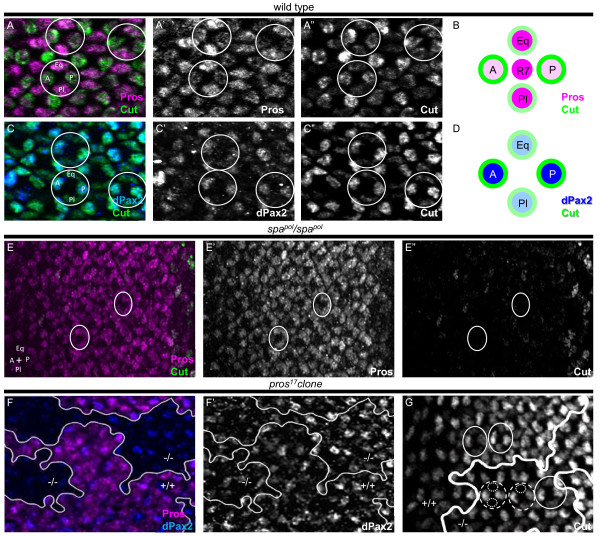
**Distinct subsets of cone cells are present in the eye imaginal disc**. **(A,C,E-G) **In wild-type late third instar eye imaginal discs, Pros (A,E,F, magenta), dPax2 (C,F, blue), and Cut (A,C,E, green; G, white) were analyzed by immunostaining. Pros is higher in the equatorial (Eq) and polar (Pl) CCs, while the expression of Cut (E,G, green) and dPax2 (G, blue) are elevated in the anterior (A) and posterior (P) CCs. **(B,D) **These expression patterns represented in diagrams. Individual ommatidia are circled; anterior is left. (E) In *spa^pol^*eye imaginal discs, Cut is mostly absent until the last few most posterior rows, while Pros expression (E, magenta; E', white) initiates in all CC precursors (circles). (F) In *pros^17^*mitotic clones, dPax2 expression (F, blue; F', white) is unchanged between *pros *mutant (Pros-negative, -/-) and wild-type (Pros-positive, magenta, +/+) tissue. (G) Cut expression is specifically delayed in Eq/Pl CCs (dotted circles) in *pros^17^*mutant tissue (-/-) (individual ommatidial CC clusters are highlighted with dashed circles), whereas in control tissue (+/+), a normal complement of four CCs is observed in comparably positioned ommatidia (solid circles).

Based on the dual requirement for Pros and dPax2 in CC development, we more closely examined the expression patterns of these transcription factors at the time that CCs are recruited in third instar eye imaginal discs. In wild-type tissue, Pros and dPax2 expression is initiated in CC precursors approximately 7 to 9 ommatidial rows anterior to the morphogenetic furrow, with Cut expression immediately following [26,28]. Surprisingly, by row 12, we find differential expression of these markers in distinct CC populations. Pros expression is higher in the equatorial and polar CCs, and weaker in the anterior and posterior CCs (Figure 2A,B), whereas dPax2 and Cut show an almost complementary expression pattern relative to Pros, with high levels in the anterior and posterior CCs, and lower levels in the equatorial and polar CCs (Figure 2C,D). These data suggest a genetic difference between anterior/posterior versus equatorial/polar CCs based on their ability to express distinct levels of Pros and dPax2/Cut.

We next examined the expression of these factors in *pros *and *dPax2 *mutants (Figure 2E-G). In *dPax2 *mutants (Figure 2E), Pros expression is initiated and maintained comparable to wild-type eyes, but Cut is almost undetectable until the last few rows of ommatidia. Therefore, although Cut is present in CCs by pupation in *dPax2 *mutants (Figure 1G) [28], CCs lacking Cut are recruited earlier in the imaginal disc (Figure 2E). This is, to our knowledge, the first report showing that CCs can be recruited in the absence of Cut. In *pros *mutant clones, dPax2 expression is similar to wild-type tissue, but Cut expression is delayed by approximately five rows, specifically in the equatorial and polar CCs (Figure 2F,G and data not shown).

Together, these data suggest that genetically distinct CCs are formed in the eye imaginal disc, that loss of Pros delays equatorial/polar CC recruitment, and that loss of dPax2 prevents Cut expression in all CCs until just prior to pupation.

### Ectopic Pros and dPax2 are sufficient to recruit extra cone cells from distinct cell populations

**Figure 3:**
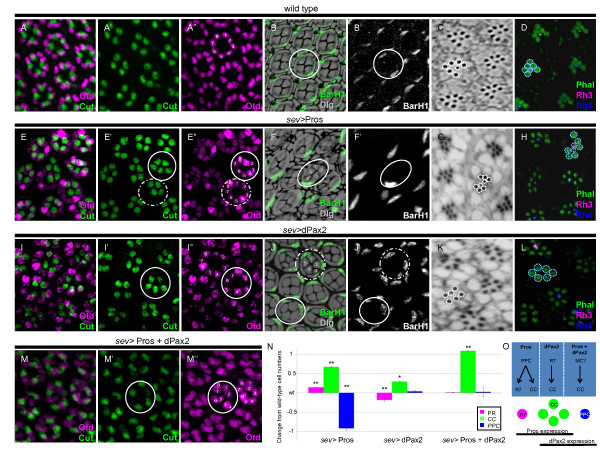
**Pros and dPax2 are sufficient to recruit cone cells, but from different cell populations**. **(A,B,E,F,I,J,M) **Retinas from 45% pupa (A,B,E,F,I,J,M) were immunostained for CC nuclei (Cut, green in (A,A',E,E',I,I',M,M')), PR nuclei (Otd, magenta in (A,A'E,E'I,I',M,M')), and PPC nuclei (BarH1, green in (B,F,J) or white in (B',F',J')), and cell outlines were revealed by Dlg staining (black in (B,F,J)). **(C,D,G,H,K,L) **Adult eyes were stained with toluidine blue (C,G,K) or immunostained (D,H,L) with the R7 opsins Rh3 (magenta) and Rh4 (blue) and fluorescently labeled phalloidin was used to mark the actin-rich apical surfaces of photoreceptors (green). **(N) **Quantification of PRs (magenta), CCs (green), and PPCs (blue). **(O) **Summary of phenotypes observed with the gain-of-function (GOF) experiments (top) and wild-type Pros and dPax2 expression patterns (bottom). MC, mystery cells [35], a potential source of ectopic CCs in the double GOF experiments. (A-D) Control pupal eyes form eight PRs (A,A'), four CCs (A,A'), and two PPCs (B,B'), and adult eyes form a trapezoid of six large outer PR rhabdomeres surrounding a smaller, central R7 PR (C) expressing Rh3 and Rh4 (D). (E,F) In *sev>*Pros pupal retinas, ectopic Cut-positive CCs are observed in ommatidia with a full complement of PRs (E, circle), and only one PPC is commonly observed (F,F', circle). Ommatidia with a complement of four CCs occasionally form an extra R7 (E, dotted circle; G,H). Ectopic CCs are also observed in *sev*>dPax2 pupal retinas (I,I'). PPCs are disorganized in *sev*>dPax2 retinas, but do not change significantly in number (J,J',N). PR number is reduced by one in *sev*>dPax2 pupal retinas (I,I') and R7s are frequently absent from adult eyes (K,L). (M) In *sev*>pros+dPax2 pupal eyes, one ectopic CC per ommatidia is frequently observed (circle), but PR and PPC numbers remain unchanged (N) (also see Additional file 1C,D). *P < 0.05, **P < 0.001. Error bars represent standard deviation.

Since Pros and dPax2 are differentially expressed in CCs, and because both factors are necessary for proper CC recruitment, we next tested whether misexpressing Pros and/or dPax2 is sufficient to drive CC formation. For this, we used a GAL4-UAS-based system to ectopically express these factors in most cell types in the imaginal disc using *sev*-GAL4 [35]. We then quantified CC, PR, and PPC numbers by immunostaining for the cell-specific markers Cut, Otd, and BarH1, respectively [36-38] (Table 1). We also analyzed the development of different PR cell types in the adult eye based on morphology and opsin expression - the R1 to R6 outer PRs arrange their actin-rich apical surfaces (rhabdomeres) as a trapezoid surrounding the central smaller R7 inner PR rhabdomere at distal sections, and R7 cells specifically express the opsin proteins Rh3 or Rh4 (Figure 3C,D) [8]. R8 cells are uniquely positioned at the proximal region of the retina, and therefore are not visible in distal retina sections.

Compared to wild-type eyes (Figure 3A), the ectopic expression of Pros (*sev>*Pros) frequently leads to ommatidia with one or two extra Cut-positive cells (Figure 3E,N). In addition, in *sev*>Pros ommatidia with a normal complement of CCs, an extra R7 is occasionally formed (Figure 3E,G,H). We also find a consistent and significant loss of one BarH1-positive PPC per ommatidia in *sev>*Pros eyes (Figure 3F,N), and this decrease is proportional to the increase in CC and R7 number we observe (Figure 3E,H,N). Ectopic expression of dPax2, on the other hand, causes 26% of ommatidia to form one additional CC, an increase that is concomitant with a reduction in R7 PRs (Figure 3I,K,L,N). We also note that a smaller population of ommatidia (11%) develops only three CCs (data not shown), consistent with previous dPax2 overexpression studies using a multimerized eye-specific enhancerto drive its expression throughout the eye [34]. Interestingly, although dPax2 is necessary for PPC formation [28], it is not sufficient for PPC development, as we do not find a significant change in PPC number in *sev>*dPax2 eyes. Moreover, in the few examples where an ectopic PPC does form in *sev>*dPax2 eyes, an extra CC is also present (Figure 3J), consistent with the fact that PPC number correlates with CC number [18,39]. Thus, ectopic dPax2 does not lead to ectopic PPC formation, but can generate ectopic CCs.

Together, these gain-of-function (GOF) experiments suggest that Pros can convert PPCs into CCs or R7s, whereas dPax2 can convert R7s into CCs. These findings also indicate that Pros and dPax2 only change fates in cells already endogenously expressing one of these two factors - Pros converts PPCs normally expressing dPax2, while dPax2 converts R7s normally expressing Pros (Figure 3O). To test whether these factors together convert cells into CCs, we co-expressed both Pros and dPax2. Like the individual GOF experiments, this co-expression consistently converts one extra cell into a CC (Figure 3M,N); in contrast, no change in PR or PPC number is observed (Table 1; Additional file 1C,D), indicating that another cell type is being converted to a CC with Pros and dPax2 co-expression. While it is currently unclear what the source of this cell is, it may correspond to a mystery cell, since these cells have been previously described to express *sev *in the imaginal disc [5,35,40]. Alternatively, it is possible that it derives from other non-specified cells in the eye that are usually eliminated by apoptosis during pupal development [41].

### Pros and dPax2 differentially regulate Ras- and Notch-dependent signaling, respectively

R7 and CC fate decisions require input from the Ras and Notch signaling pathways, and Pros and dPax2 are both direct downstream targets of these pathways [26,27,29,30]. Thus, these transcription factors are likely candidates for mediating downstream events and/or feedback regulatory control of these pathways. To test this hypothesis, we analyzed *pros *and *dPax2 *loss-of-function (LOF) mutants for factors that lie downstream of Ras (for example, activated MAPK and Yan) and Notch (for example, E(spl) and Delta).

**Figure 4:**
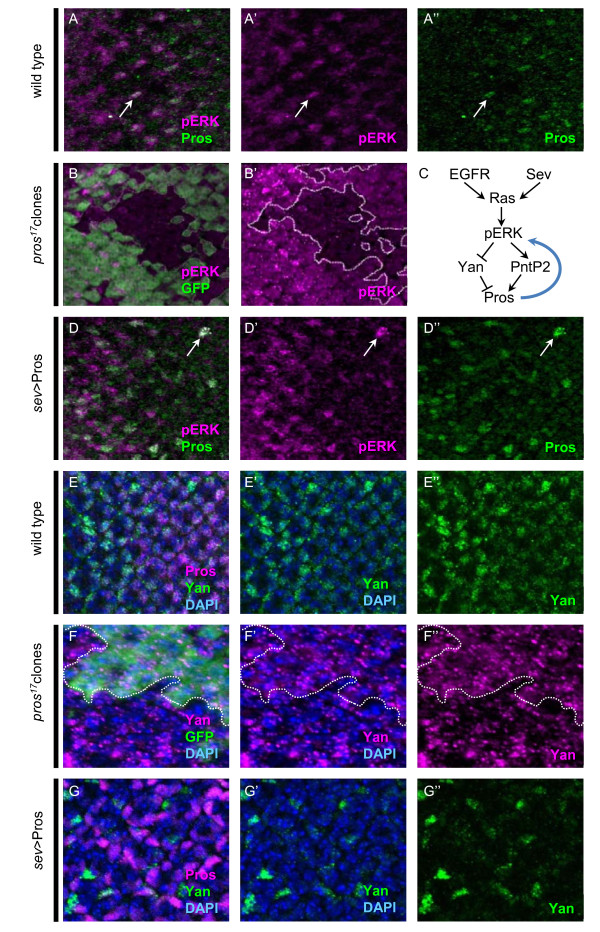
**Pros is necessary and sufficient for high pERK levels**. **(A,B,D-G) **Eye imaginal discs from control (A,E), *pros^1^*^7^mitotic clones (B,F) and *sev*>Pros eyes (D,G) were immunostained for Pros (A,B, green; E,G, magenta), pERK (A,B,D, magenta), and Yan (E,G, green; F, magenta). GFP was used to mark wild-type (versus *pros^1^*^7^mutant) tissue (B,F, green), and nuclei were visualized with DAPI (E,F,G, blue). **(C) **A diagram representing Pros regulation by Ras/MAPK signaling previously reported [26,27,29], and the positive feedback onto pERK described here. In optical sections at the level of the R7 and CCs, high Pros expression correlates with high pERK expression (A,C, arrows), whereas a cell autonomous reduction in pERK is observed in *pros *mutant tissue (B, non-GFP positive cells). No change in pERK is observed in more basal optical sections where Pros is not expressed (data not shown). (E) In wild-type imaginal discs, Yan expression (green) becomes reduced as Pros expression (magenta) increases in specified CCs. (F) In *pros *mutants, Yan (magenta) is only present in nuclei in GFP-negative (that is, *pros*-negative) tissue, whereas it is both nuclear and cytoplasmic in surrounding GFP-positive wild-type tissue (green). (G) In *sev*>Pros imaginal discs, Yan (green) is reduced throughout the disc, being almost undetectable in cells expressing particularly high levels of Pros (magenta).

Consistent with Pros being strongly activated by high Ras/MAPK levels provided by EGFR and Sev signaling, wild-type ommatidia show a strong correlation between high levels of Pros expression and activated MAPK (nuclear pERK; Figure 4A). Interestingly, in *pros *mutant clones, we observe a dramatic cell autonomous decrease in pERK levels (Figure 4B), while cytoplasmic (un-activated) ERK levels appear unchanged (data not shown). Additionally, cells overexpressing Pros show increased nuclear pERK levels (Figure 4D, arrow). These data suggest that Pros is not only a downstream target of pERK activity, but also plays a positive feedback role to maintain high pERK levels (Figure 4C).

Since pERK phosphorylates Yan and blocks the nuclear accumulation of this ETS-related transcriptional repressor [42], as a further test that Pros effects pERK levels, we next examined Yan expression in *pros *LOF and GOF tissues. Reflective of the fact that Yan directly represses Pros expression [27], Yan levels decrease at the time that Pros levels increase in wild-type tissue (Figure 4E). However, in *pros *mutant clones, nuclear Yan levels increase (Figure 4F), and in *sev*>Pros tissue, Yan levels decrease (Figure 4G). This provides additional support for a role for Pros in up-regulating pERK activity. We also functionally tested a role for Pros in up-regulating pERK activity by taking advantage of the fact that R7 specification fails in *sev *flies due to insufficient Ras/pERK signaling [2,10,16,24]. We reasoned that if Pros is sufficient to up-regulate pERK, then misexpressing Pros in *sev *mutants should rescue R7 differentiation. Indeed, *sev*; *sev>*Pros flies develop an R7 in >90% of ommatidia, whereas no R7 forms in *sev *mutants alone (Table 1; Additional file 1E,F).

**Figure 5:**
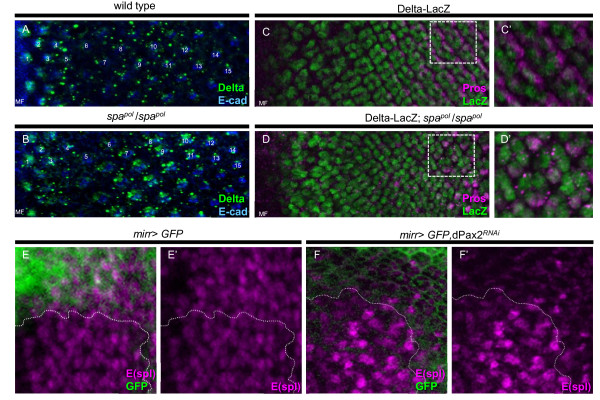
**dPax2 effects Delta/Notch signaling**. **(A-F) **Eye imaginal discs from control (A,C), *spa^pol^*mutants (B,C), *mirr*>GFP (E), and *mirr*>GFP,dPax2^RNAi ^were immunostained for Delta (A,B, green), E-cadherin (A,B, blue), LacZ (C,D, green), Pros (C,D, magenta), GFP (E,F, green), or E(spl) (E,F, magenta). All discs are oriented with anterior left. In wild-type discs, Delta expression is high in the first four rows of ommatidia after the morphogenetic furrow (MF) and then decreases thereafter (A). In *spa^pol^*mutants, however, Delta is up-regulated again by row 7. Delta-LacZ expression in wild-type discs is down-regulated at the more posterior rows of the disc and do not co-localize with Pros in CC precursors (C), whereas in *spa^pol ^*mutants, Delta-LacZ expression is maintained at high levels throughout the disc and co-localizes with Pros in the most posterior rows (D), indicating that dPax2 transcriptionally represses Delta expression in CC precursors. E(spl) (E,F) is equally expressed when a UAS-GFP transgene is misexpressed in the dorsal half of the eye imaginal disc with *mirr*-GAL4 (E), but is significantly reduced where UAS-dPax2^RNAi^/UAS-GFP are co-expressed (F), revealing that dPax2 is important for maintaining high Notch activity.

We next analyzed *dPax2 *mutants. In contrast to *pros *mutants, we do not detect obvious changes in Ras signaling molecules in *spa^pol^*or *sev*>*dPax2^RNAi^*flies (data not shown). However, we do find a significant increase in Delta expression in the seventh row of ommatidia, the same row of ommatidia that dPax2 expression is normally activated [28], and the row before normal Delta onset in the anterior and posterior CCs [43] (Figure 5A versus 5B). Moreover, co-staining of Pros and a *Delta*-LacZ reporter line reveals a cell-autonomous up-regulation of Delta gene expression in *spa^pol^*mutant CC precursors compared with controls (Figure 5C,D). These findings collectively indicate that dPax2 is important for transcriptionally repressing *Delta *during CC recruitment.

In many systems, Delta is repressed in Notch signal-receiving cells to prevent receptor-ligand co-expression and facilitate unidirectional cell-cell signaling [20]. Since Delta expression is up-regulated in *dPax2 *mutants, we also analyzed Notch activation in these mutants by examining the expression of another Notch target gene, E(spl) [44]. This shows that E(spl) expression is significantly and cell-autonomously reduced in RNAi-mediated dPax2 knockdown tissue compared with control tissue (Figure 5E,F). Due to the extensive feedback between Notch and Delta, determining whether dPax2 is necessary for directly repressing Delta and/or promoting Notch activity requires further tests. Nevertheless, our experiments provide evidence that dPax2 controls at least some aspects of Notch signaling, likely by repressing Delta, and that Pros helps mediate downstream processes for Sev and/or EGF signaling by maintaining increased pERK levels.

### Pros and dPax2 combinatorially control neuronal versus non-neuronal cell fate decisions

**Figure 6:**
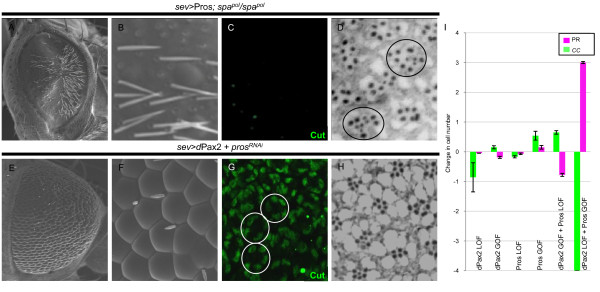
**dPax and Pros control the neuronal to non-neuronal switch in the R7 equivalence group**. **(A-D) **In eyes overexpressing Pros in the absence of dPax2 (*sev>*Pros; *spa^pol^*eyes), lenses are almost absent by SEM (A,B), no Cut-positive CCs are observed (C, green), and R7 number is significantly increased, with three to four often present in individual ommatidia (D, circles). **(E-H) **In eyes overexpressing dPax2 in the absence of Pros (*sev>*dPax2+*pros^RNAi^*eyes), lens formation is mildly disrupted (E,F), an average of five CCs/ommatidia are formed (G, circles), and R7 PRs are rarely observed (H). **(I) **Quantification of PR (magenta) and CC (green) numbers in *pros *and *dPax2 *LOF and GOF experiments (see Table 1 for values, and Materials and methods for specific genotypes). Error bars represent standard deviation

Notch signaling is commonly associated with non-neuronal fate decisions, and in the fly eye, increased Ras activation functions as a pro-neuronal factor, able to transform epithelial CCs into a neuronal R7. Based on our findings that Pros and dPax2 individually control distinct aspects of Ras and Notch signaling, respectively, and that neither factor alone efficiently changes cell fates, we asked whether these factors oppose one another in the R7 equivalence group to affect neuronal (R7) versus non-neuronal (CC) cell fate decisions. For testing this hypothesis, we overexpressed Pros in *dPax2 *mutants, and conversely, overexpressed dPax2 in *pros *mutants. Pros overexpression in *dPax2 *mutants (*sev*>*Pros *+ *dPax2^RNAi^*or sev*>Pros*; *spa^pol^*) results in ommatidia with up to four R7 cells, with an average of 3.0 (Figure 6D,I; Table 1). In addition, no CCs are detected and adult lenses fail to form (Figure 6A-C). Comparatively, overexpressing Pros in cells maintaining endogenous *dPax2 *produces only 1.15 R7s/ommatidia and increases CC number (Figures 3E,N and Figure 6D; Table 1). Thus, these data support a model in which dPax2 normally blocks the ability of Pros to convert R7s to CCs. Conversely, removing *pros *while misexpressing dPax2 (*sev*-GAL4>*dPax2 *+ *Pros^RNAi^*) significantly increases dPax2's ability to transform R7s into CCs, with the majority of ommatidia forming five CCs and lacking R7s (Figure 6G-I; compare dPax2 GOF with dPax2 GOF + pros LOF in Figure 6I and Table 1). Mild eye roughening is also observed (Figure 6E,F), consistent with the presence of extra CCs. Combined, these experiments suggest that Pros and dPax2 function antagonistically to control neuronal versus non-neuronal fate based on their ability to regulate Ras and Notch signaling, respectively.

## Discussion

Here, we present evidence that Pros and dPax2 together specify neuronal versus non-neuronal fates by combined feedback into the Ras/MAPK and Delta/Notch signaling pathways, respectively. Specifically, we demonstrate that Pros and dPax2 are differentially expressed in distinct CC subpopulations, and synergistically contribute to CC recruitment and lens formation. Moreover, we show that within the R7 equivalence group, Pros is required for obtaining the high pERK levels necessary for sensory (R7) cell fate determination, whereas dPax2 is critical for repressing Delta expression in response to Notch to block sensory cell specification. Thus, in one context (that is, CC recruitment), Pros and dPax2 function cooperatively, whereas in another (that is, R7 versus CC fate choice), they function antagonistically. These studies ultimately reveal that Pros and dPax2 are mediators of the same signaling pathways that initiate their expression, and that cells previously considered equivalent are instead differentially sensitized to EGF and Notch signaling based on the expression levels of two distinct transcription factors. Interestingly, cell lineages in many other sensory organs also make use of reiterative Notch and Ras signaling, express dynamic levels of dPax2 and Pros, and require dPax2 and Pros for distinct cell fate decisions [45-48]. Thus, Pros and dPax2 are likely to perform similar functions in other regions of the *Drosophila *nervous system. Moreover, as we discuss later, these functions may be evolutionarily conserved in other animals and developmental contexts.

### Neuronal versus non-neuronal cell fate decisions by the combinatorial actions of Pros and dPax2

One of the best-studied systems related to breaking 'equivalence' in a group of cells is lateral inhibition during neurogenesis. In this process, a field of neuroepithelial cells initially expresses uniform and low levels of a member of the proneural family of basic helix-loop-helix (bHLH) transcription factors. bHLHs activate Delta, whereas Notch activation suppresses bHLH and Delta expression. Thus, through a feedback system between Notch and Delta, bHLHs and Delta become progressively restricted to one cell within the proneural field. This cell differentiates into a neuron, whereas its neighboring cells receive high levels of anti-neural Notch signaling and remain epithelial [20].

**Figure 7:**
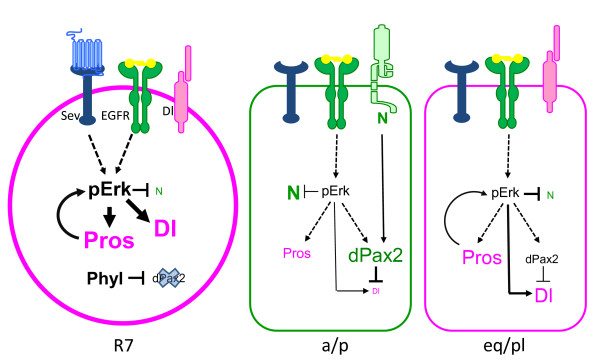
**Proposed model for the roles of Pros and dPax2 during cell fate decisions in the R7 equivalence group**. Model depicting the outcome of Ras/MAPK and Notch (N/Delta (Dl signaling in the R7, anterior (a)/posterior (p) CCs, and equatorial (eq)/polar (pl) CCs based on the differences in Pros and dPax2 observed in these different cell populations. Phyllopod (Phyl) has been previously suggested to prevent dPax2 expression in the presumptive R7 [34]. In this model, the a/p CCs would be dominated by dPax2/Cut/Notch activity, whereas the eq/pl CCs would be dominated by Pros/pERK/Delta. Based on our LOF analysis, we propose that a/p and eq/pl CCs represent two distinct CCs that are inter-convertible. Moreover, in *pros *mutants, we postulate that four 'a/p' CCs form, whereas in dPax2 mutants, 'eq/pl' CCs form.

In a similar manner, we propose that the R7 equivalence group is much like a proneural field, and that Pros and dPax2 specify neuronal R7 fate versus non-neuronal CC fate by controlling Notch and Delta levels. Consistent with this model, dPax2: a) is expressed only in non-neuronal cells of the equivalence group; b) transforms the R7 PR into a non-neuronal CC when overexpressed (Figure 3I-L); and c) suppresses Delta expression and/or promotes Notch activity (Figure 5A-D), a combination usually associated with non-neuronal fates [20]. Together, these findings support a function for dPax2 in specifying non-neuronal CC fate within the R7 equivalence group. Pros, on the other hand, is frequently associated with neural fates in the embryonic nervous system [49,50] and is essential for maintaining high pERK levels required for R7 neuronal fate choices in the R7 equivalence group (Figure 4). Importantly, recent studies have demonstrated that pERK indirectly activates Delta expression and degrades the intracellular domain of Notch in the R7 equivalence group [18,51]. Consistent with this and our finding that Pros up-regulates pERK levels, Delta expression is reduced in *pros *mutant clones compared to surrounding wild-type tissue (Additional file 2). The ability of Pros to indirectly affect Notch and/or Delta also helps to explain the non-cell-autonomous effects on CC numbers we observe at *pros *clone borders (Additional file 1A,B), since an imbalance in cell-cell dependent Notch signaling should primarily affect cells in highly mosaic tissue. Hence, we propose that Pros and dPax2 combinatorially influence R7 versus CC fate, in large part by controlling Notch signaling levels (Figure 7): dPax2 suppresses Delta, thereby enhancing Notch receptor-mediated anti-neuronal signals, whereas Pros activates Delta and suppresses Notch receptor activation indirectly through pERK, thereby promoting neural fates. Current efforts are focused on defining the precise mechanisms of how Pros and dPax2 exert their effects on pERK and Delta. Since pERK is regulated post-translationally, and we do not find detectable changes in unmodified ERK levels, it is unlikely that Pros affects pERK levels transcriptionally. However, since Pros can function as either an activator or a repressor [32,52], and many transcriptional targets can feed back into the Ras/MAPK pathway (for example, [53]), identifying the transcriptional target(s) of Pros represents somewhat of a challenge. dPax2, on the other hand, appears to transcriptionally repress Delta, either directly or through regulation of Notch/E(spl). In favor of direct regulation, potential dPax2 sites do exist in non-coding regions of the Delta locus (MCP and TC, unpublished). Additionally, since dPax2 affects Cut expression, and in the wing disc, Cut has been postulated to control Delta expression [54], it is possible that dPax2 and/or Cut are involved in regulating Delta expression. Thus, future studies focused on testing the ability of these factors to control Delta gene expression may provide important mechanistic insight into how the Notch pathway is controlled in various tissues.

### Becoming non-equivalent within the R7 equivalence group by Pros and dPax2 expression

#### R7 versus cone cells

This study suggests that at least two subclasses of CCs are specified in the eye based on differing relative expression levels of Pros and dPax2/Cut. Importantly, the eye-regulatory enhancers for both Pros and dPax2 give a 'salt-and-pepper' expression pattern to reporter genes [29,31] (MCP and TC, unpublished), suggesting that these differences are controlled transcriptionally. Given our findings that Pros and dPax2 feedback into the same pathways known to control their expression, these transcription factors may also have some auto-regulatory functions in this system. Notably, however, even though Pros and dPax2 expression requires input from Ras/pERK and Notch-dependent signaling, and both pathways are required for R7 and CC fate, Pros is expressed in all five cells of the R7 equivalence group whereas dPax2 is restricted to CC precursors. This observation raises the possibility that Boss-Sevenless signaling not only activates Pros expression, but may also actively suppress dPax2 expression in the presumptive R7. Such a scenario would also help explain recent findings by Swanson and colleagues [31] showing that disrupting the dPax2 CC enhancer often causes inappropriate expression in PRs - perhaps these artificial enhancers lack or disrupt Sev-dependent repression elements. Since expressing an activated Sev receptor leads to a higher average number of ectopic R7 cells than expressing constitutively active Ras in the same cells (2.8 per ommatidia with *sev*-Sev^S11^) versus approximately 0.65 for *sev*-RasV^12 ^(Table 1) [10,17,24,55], and because misexpressing Pros only weakly converts CCs into R7 cells unless dPax2 is also removed (Figure 6), it is unlikely that repression of dPax2 in the presumptive R7 is mediated through Ras/pERK. Thus, we propose that while Pros mediates the Ras output of the Sev receptor, another factor activated in the presumptive R7, possibly Phyllopod [34], suppresses dPax2 in R7 cells. These two events together would then help define differences between the R7 and CC precursors within the R7 equivalence group.

#### Cone cell subtypes

Our findings that different CC subtypes exist during their early recruitment provide a useful paradigm for interpreting previous experiments focused on R7 versus CC specification. For instance, despite the widely held paradigm that EGF and Notch are 'equivalent' in all cells within the R7 equivalence group, the majority of mutants tested to support this model generally only affect a subset of cells [10,17,19,21,23,26,30,55-57]. One explanation for this is that these different mutants differentially participate in the formation of distinct CCs. Thus, re-examining these mutant phenotypes may help define other targets associated with CC subtype specification and/or differentiation and help us understand the biological relevance of having two CC subtypes.

### Potential parallel functions for Pros/Prox1 and dPax2/Pax2 in other systems

To date, whether Pax2 or Pros are involved in regulating Notch or MAPK signaling in other systems has not been directly tested. However, Pros has been implicated in regulating Ras signaling via the EGFR ligand Vein in *Drosophila *glioblast formation [58], and several components within the Ras and Notch signaling pathways have been identified as potential *pros *targets by microarray analysis [59,60]. Similarly, the vertebrate ortholog of Pros, Prox1, is known to act downstream of tyrosine kinase-dependent Ras signaling in differentiating lens fiber cells [61,62]. In addition, Notch signaling has recently been implicated in vertebrate lens development, and the required Notch ligand is supplied by both cell populations that express Prox1: epithelial lens progenitors and differentiating primary fiber cells [63-65]. Thus, it is possible that the ability of Pros to affect Ras and Notch signaling may be evolutionarily conserved. Further supporting this hypothesis, Prox1 has recently been shown to reduce Notch activity to induce neurogenesis [66]. Likewise, similar to *Drosophila *Pax2, vertebrate Pax2 is frequently a target of Notch signaling in sensory systems, including the inner ear, and has been proposed to mediate at least some of the Notch-dependent functions important for hair cell development [67]. Pax2 and Notch signaling are also critical regulators of kidney development, and both are potential therapeutic targets in many renal cancers [68-72]. Therefore, we are optimistic that the present findings will provide the theoretical framework and a tractable genetic system for advancing our understanding of developmental processes in a wide range of biological systems.

## Materials and methods

### Generation of RNAi lines

Two UAS-*dPax2 *RNAi lines were generated using the following primers against the Sv cDNA cpx1 [45]: forward 1, CTGA*GAATTC*ATGCTTATAATGGATATACAGACATCG (*Eco*RI), reverse 1, CTGA*TCTAGA*GTTGTATTCCTAATATTCCATTTATGC (*Xba*I); forward 2, CTGA*GAATTC*AATTGTAAGGAATAAAGCCGCCGAG (*Eco*RI), reverse 2, CTGA*TCTAGA*GTTGTATTCCTAATATTCCATTTATGC (*Xba*I).

Inverted repeats were ligated with *Eco*R1, and then cloned into pMF3 (Vienna *Drosophila *RNAi Center) with *Xba*I. A UAS-Pros RNAi line was generated by amplifying from ProsS cDNA [32] using the primers AA*GGATCC*CGGCTGCCATGTTCCAGGCGC (*Bam*HI) and CC*TGCGCA*ATGGCGCTTCTTCTTTGGTGTC (*Mlu*I). (Italics represent the restriction enzyme sites that appear in parenthesese following the sequence. Inverted repeats were ligated with *Mlu*I, subcloned with *Bam*HI into pBSIIKS(-) in Sure Cells (Invitrogen, Carlsbad, CA, USA), and cloned into pUAST [73] using *Xba*I and *Xho*I. Transgenic lines were generated in *yw^67^*flies using standard procedures (Rainbow Transgenics, Camarillo, CA, USA). Multiple lines for each construct were tested and those phenocopying *spa^pol^*or *pros^17^*mutants were retained for further analysis.

### Fly genetics

FRT82B*ubi*-GFPnls, RpS3/TM6B, FRT82B*ubi*-GFP/TM6B, sev, *spa^pol^*, Dl-LacZ, *sev-*Ras^v12 ^*, sev*-Gal4, *mirr*-GAL4, and UAS-CD8::GFP flies were from the Bloomington Stock Center. Other lines were: UAS-*prosS *and FRT82B-*pros^17^*[32], *ey^flp3.5^*(provided by Claude Desplan), UAS-*dPax2 *[45], and UAS-Pros^KKRNAi ^(Vienna *Drosophila *RNAi Center). *prospero *mitotic clones were analyzed in *ey^flp3.5^*; Sp/CyO; FRT82ubi-GFP/FRT82B-*pros*^17 ^flies, whereas Minute clones to generate eyes almost entirely mutant for *pros *were generated from *ey^flp3.5^*; Sp/CyO; FRT82*ubi*-GFPnls, RpS3/FRT82B-*pros*^17 ^flies. Other genotypes were: *yw^67^*; Sp/CyO; TM2/TM6B ('wild-type'), *yw^67^*; Sp/CyO; TM2/TM6B; *spa^pol^*/*spa^pol^*(*d*Pax2 LOF), *yw^67^*; Sp/CyO; FRT82B-*pros*^17^/TM6B; *spa^pol^*/*spa^pol^*(*pros/+*, *dPax2 *LOF), UAS-*d*Pax2^RNAi^; sev-GAL4/CyO; UAS-Pros ^RNAi^/TM6B (*sev>dPax2^RNAi ^+ Pros^RNAi^*), *yw^67^*; *sev*-Gal4; UAS-Pros/TM6B (*sev>pros*), *yw^67^*; *sev*-Gal4; UAS-*dPax2/TM6B *(*sev>dPax2*), *yw^67^*; *sev*-Gal4; UAS-*dPax2*/UAS-Pros (*sev>*Pros/dPax2), *sev*; Sp/CyO; TM2/TM6B (*sev*), *sev*; *sev*-Gal4/CyO; UAS-Pros/TM6B (*sev*; *sev>*Pros), *yw^67^*; *sev*-Gal4/CyO; UAS-Pros/TM6B; *spa^pol^*/*spa^pol^*(Pros GOF, dPax2 LOF), and *yw^67^*; Sp/*sev*-Ras^v12^, CyO; TM2/TM6B (*sev*-Ras^v12^), *yw^67^*; sev-GAL4/UAS-pros^KKRNAi^; UAS-dPax2/TM2 (*sev>dPax2 + Pros^RNAi^*), *yw^67^*; UAS-CD8::GFP; *mirr*-GAL4 (*mirr*>GFP), UAS-*d*Pax2^RNAi^; UAS-CD8::GFP/Sp (or CyO); *mirr*-GAL4/TM2 (*mirr*>*GFP,d*Pax2^RNAi^). Flies were raised on standard cornmeal/yeast/molasses/agar media at *25*°C.

### Antibody production

Primary antibodies were generated by Cocalico Biologicals (Reamstown, PA, USA) against denatured His-tagged proteins produced from pET-28a (+) as previously described [74]. Rat anti-BarH1 was created against a full-length protein made from the BarH1 cDNA present in pGEX-4T-*BarH1 *[38], guinea pig anti-Pros was produced against amino acids 399 to 972 from a *Sac*I fragment from ProsS [75], and anti-dPax2 antibodies were produced against amino acids 308 to 512 from a *Bam*H1/*Eco*RI fragment from pGEX-*dPax2 *[28]. Specificity was determined by comparison with previously described staining patterns, and in *spa^pol^*or *pros^17^*mutants. Guinea pig anti-Pros immunolocalization was further confirmed with two previously described antibodies: mouse MR1A against amino acids 1,196 to 1,320 [76], and rabbit 89E against amino acids 409 to 438 [50].

### Histology and microscopy

Adult lenses were visualized by SEM using anesthetized flies mounted on carbon tabs and directly analyzing them with a Hitachi S-3400N. For thin sections, eyes were fixed for 15 minutes in 4% paraformaldehyde/phosphate-buffered saline, washed twice with 0.1% Triton X/phosphate-buffered saline (PBT), serially dehydrated in ethanol, incubated in 1:1 ethanol/LR-White resin (Electron Microscopy Sciences, Hatfield, PA, USA) for 1 hour and 100% resin for 1 hour, then polymerized with one drop per milliliter of accelerator. Sections (2 μm) were dried for 15 minutes on a slide warmer and stained with 1% toluidine blue for 10 minutes and mounted in Entellan (EMS), or rehydrated in PBT overnight followed by antibody staining and mounting as previously described [74]. Imaginal discs and retinas dissected from pupa 45 hours after puparium formation (45% pupation) were immunostained as previously described for whole mount adult retinas [74]. Antibodies were from the Developmental Studies Hybridoma Bank unless indicated otherwise, and diluted as follows: Cut (mouse, 1:100), Pros (rabbit [50], 1:1,000), Pros (mouse, 1:10), Pros (guinea pig, this paper, 1:1,500), dPax2 (rabbit [28], 1:50; rabbit, this paper, 1:1500; guinea pig, this paper, 1;1,500), Elav (mouse or rat, 1:200), Eya (mouse, 1:50), BarH1 (rat, this paper, 1:200; rabbit [38], 1:50), Dlg (mouse, 1:50), E-cadherin (rat, 1:20; rabbit, Santa Cruz Biotechnology, Inc., Santa Cruz, CA, USA, 1:50), GFP (chicken, Abcam, Cambridge, MA, USA, 1:500), β-gal (chicken, Sigma-Aldrich, St. Louis, MO, USA, 1:1,000), Otd (guinea pig [74], 1:750), Rh3 (chicken [32], 1:40), Rh4 (rabbit, gift from C Zuker/N Colley, 1:150), Delta (mouse, 1:50), E(spl) (mouse [44], 1:1), pERK (rabbit, Sigma-Aldrich, 1:20), m(Espl) (1:2 [44]) and N-Cad (rat, 1:20). Secondary antibodies were conjugated to Alexa Fluor 488, 555, 647 and 750 nm (goat, Invitrogen) or Cy2, 3 or 5 (donkey, Jackson Immunoresearch, West Grove, PA, USA), and diluted 1:500. Polymerized actin was detected using AlexFluor 488-conjugated phalloidin (Invitrogen), which was added to the secondary antibody dilutions at 1:20 according to the manufacturer's suggestion. Samples were imaged with a Zeiss Apotome, deconvolved with Axiovision 4.6 or Zeiss LSM 700 confocal and processed in Adobe Photoshop 7.0.

### Cell type quantification

Flies were raised at 25°C, pupal retinas were dissected at 45 hours after puparium formation (45% pupation), and samples were stained with Otd, Cut, and BarH1 to identify PRs, CCs, and PPCs, respectively. Individual ommatidia were defined by co-staining with either E-cadherin or Dlg. Counting from a minimum of 100 ommatidia from at least for separate mutant eyes was performed, and compared to wild-type values using one-way ANOVA (Microsoft Excel; StatPlus). For *pros *center versus border clonal analysis, clone borders were defined as being within at least one ommatidium of any GFP-expressing cell, while clone centers were at least two ommatidial spaces away from any GFP-expressing cell.

## Abbreviations

bHLH: basic helix-loop-helix; CC: cone cell; EGF: epidermal growth factor; EGFR: epidermal growth factor receptor; GFP: green fluorescent protein; GOF: gain-of-function; LOF: loss-of-function; MAPK: mitogen-activated protein kinase; PPC: primary pigment cell; PR: photoreceptor; Pros: Prospero; RNAi: RNA interference; SEM: scanning electron microscopy.

## Competing interests

The authors declare that they have no competing interests.

## Authors' contributions

MCP helped design the study, carried out the molecular genetic studies and statistical analysis, and drafted the manuscript, SLW initiated the molecular genetic studies on Pros, YF participated in molecular genetic studies of Pros and dPax2, BX generated antibody reagents, DLK generated the dPax2 RNAi constructs, BG participated in the design of the study, and TAC helped in the design, coordination, and analysis of the study and completed the writing of the manuscript. All authors read and approved the final manuscript.

## Supplementary Material

Additional file 1 **Pros and dPax2 functions in photoreceptors, cone cells, and primary pigment cell formation**. **(A,B) **Pupal retinas 45 hours after puparium formationwith *pros^17^*clonal tissue were co-stained with Dlg (black), BarH1 (green), and GFP (magenta). CCs and PPCs are pseudocolored in green and blue in (A',B') to help visualize the cell types. Wild-type and *pros *heterozygous tissue in (B) is GFP-positive. In the center of *pros *clones (A), four CCs and two PPCs normally form, whereas at clone borders or in highly mosaic tissue (B), CCs are frequently lost and ectopic PPCs are readily observed, indicating that *pros *has non cell-autonomous functions during CC recruitment. **(C,D) **Pupal retinas 45 hours after puparium formation stained with E-cadherin (white) and pseudocolored with blue to highlight PPCs from wild-type eyes (C) and eyes misexpressing Pros and dPax2 (sev>dPax2+Pros) (D) reveal that two PPCs regularly form in both cases. **(E,F) **Plastic sections at the R7 layer of adult eyes from *sev^- ^*(E), and *sev^-^*; *sev>*Pros (F) show that the small R7 rhabdomeres are absent in *sev *eyes, but are present in almost all ommatidia with the addition of Pros. Click here for file

Additional file 2 **Pros affects Delta expression in the eye imaginal disc**. Mitotic clones of *pros^17^*were analyzed in late third instar eye imaginal discs for Delta expression (green). *pros *clones are revealed by co-staining with Pros (magenta). Delta levels are decreased in *pros *mutant tissue compared to surrounding wild-type tissue, supporting evidence that pERK can indirectly affect Delta expression [18,19,51]. Click here for file
