# Supplementary material for: Prospero and Pax2 combinatorially control neural cell fate decisions by modulating Ras- and Notch-dependent signaling
Source: Neural Dev. 2011 May 3;6:20. doi: 10.1186/1749-8104-6-20 (PMC3123624; doi:10.1186/1749-8104-6-20)
Supplement: Additional file 1 — Pros and dPax2 functions in photoreceptors, cone cells, and primary pigment cell formation. (A,B) Pupal retinas 45 hours after puparium formationwith pros17clonal tissue were co-stained with Dlg (black), BarH1 (green), and GFP (magenta). CCs and PPCs are pseudocolored in green and blue in (A',B') to help visualize the cell types. Wild-type and pros heterozygous tissue in (B) is GFP-positive. In the center of pros clones (A), four CCs and two PPCs normally form, whereas at clone borders or in highly mosaic tissue (B), CCs are frequently lost and ectopic PPCs are readily observed, indicating that pros has non cell-autonomous functions during CC recruitment. (C,D) Pupal retinas 45 hours after puparium formation stained with E-cadherin (white) and pseudocolored with blue to highlight PPCs from wild-type eyes (C) and eyes misexpressing Pros and dPax2 (sev>dPax2+Pros) (D) reveal that two PPCs regularly form in both cases. (E,F) Plastic sections at the R7 layer of adult eyes from sev- (E), and sev-; sev>Pros (F) show that the small R7 rhabdomeres are absent in sev eyes, but are present in almost all ommatidia with the addition of Pros. [file 1749-8104-6-20-S1.PPT]

## Slide 1
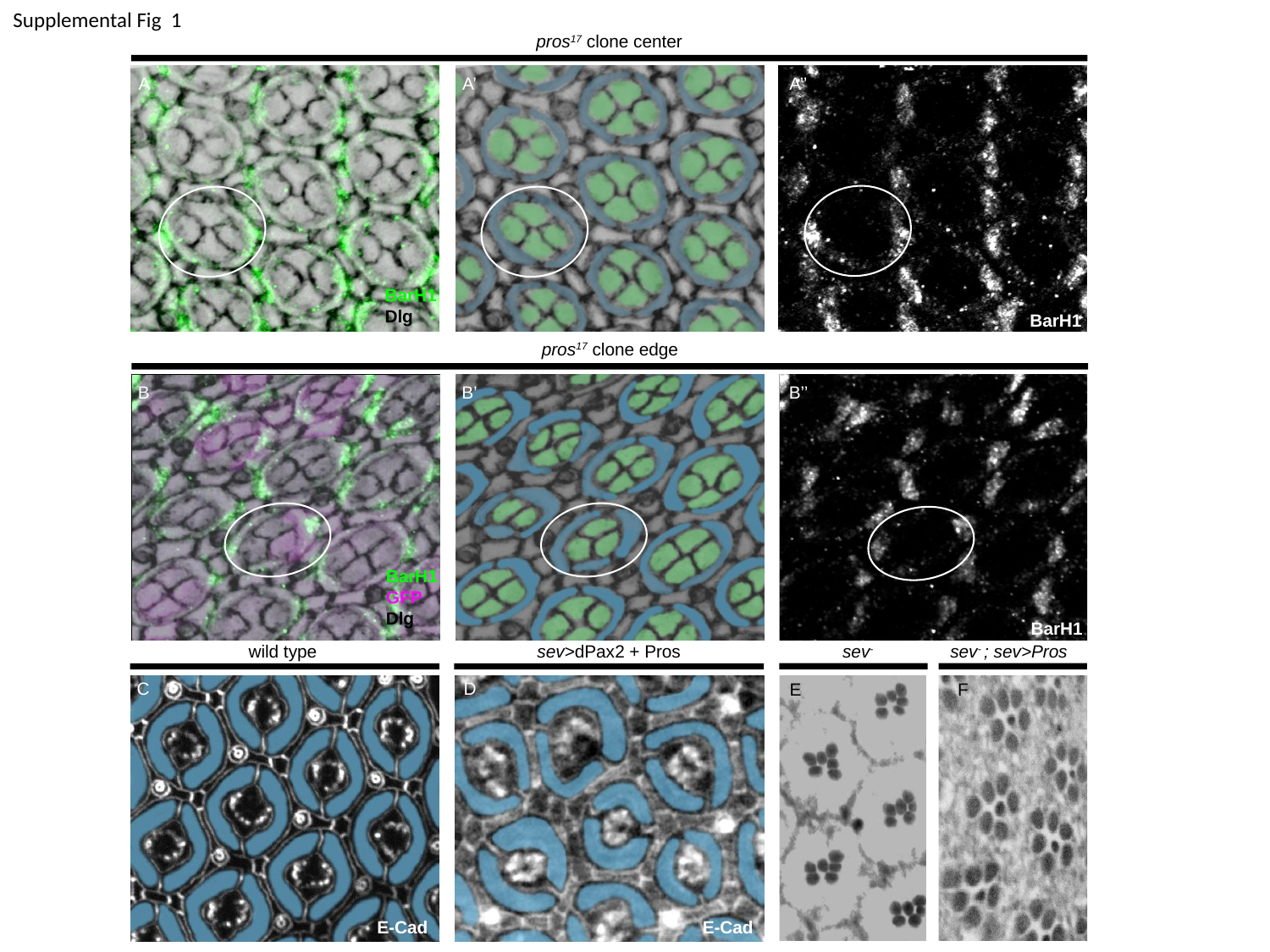

Supplemental Fig 1
pros17 clone center
BarH1
A
A’
A’’
BarH1
Dlg
pros17 clone edge
B
B’
B’’
BarH1
GFP
Dlg
BarH1
wild type
sev>dPax2 + Pros
sev-
sev- ; sev>Pros
C
D
E
F
E-Cad
E-Cad
