# Supplementary figures and images for: Prospero and Pax2 combinatorially control neural cell fate decisions by modulating Ras- and Notch-dependent signaling
Source: Neural Dev. 2011 May 3;6:20. doi: 10.1186/1749-8104-6-20 (PMC3123624; doi:10.1186/1749-8104-6-20)

## Slide 1
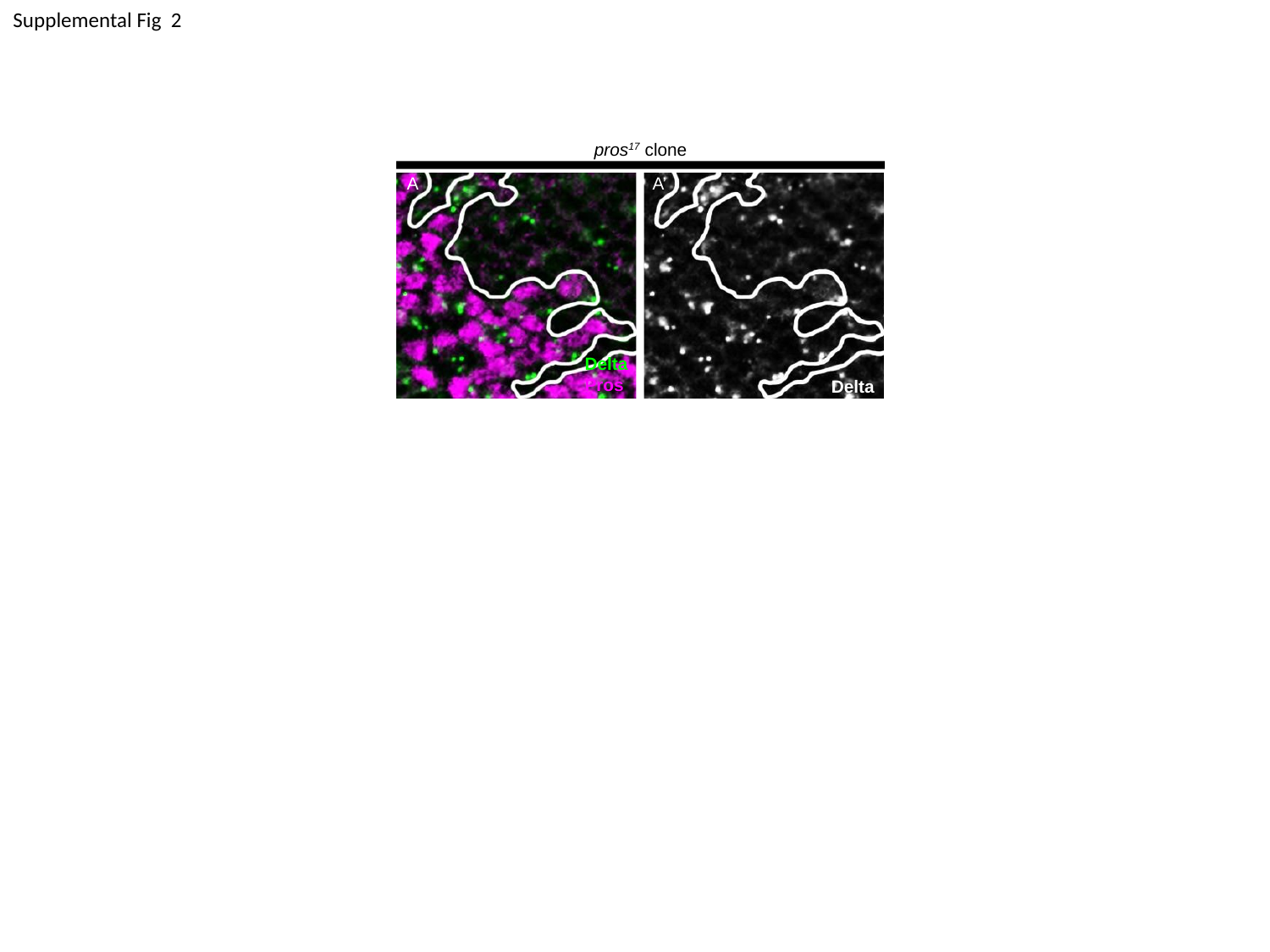

Supplemental Fig 2
pros17 clone
A
A’
Delta
Pros
Delta

Supplement: Additional file 2 — Pros affects Delta expression in the eye imaginal disc. Mitotic clones of pros17were analyzed in late third instar eye imaginal discs for Delta expression (green). pros clones are revealed by co-staining with Pros (magenta). Delta levels are decreased in pros mutant tissue compared to surrounding wild-type tissue, supporting evidence that pERK can indirectly affect Delta expression [18,19,51]. [file 1749-8104-6-20-S2.PPT]
